# Inhibition of endogenous NGF degradation induces mechanical allodynia and thermal hyperalgesia in rats

**DOI:** 10.1186/1744-8069-9-37

**Published:** 2013-07-29

**Authors:** Maria Osikowicz, Geraldine Longo, Simon Allard, A Claudio Cuello, Alfredo Ribeiro-da-Silva

**Affiliations:** 1 Department of Pharmacology and Therapeutics, McGill University, 3655 Prom Sir-William-Osler, Montreal, QC H3G 1Y6, Canada; 2 Department of Anatomy and Cell Biology, McGill University, Montreal, QC H3A 2B2, Canada; 3 Department of Neurology and Neurosurgery, McGill University, Montreal, QC H3A 2B4, Canada

**Keywords:** Matrix metalloproteinase, Nerve growth factor, Allodynia, Hyperalgesia, Sympathetic sprouting

## Abstract

**Background:**

We have previously shown a sprouting of sympathetic fibers into the upper dermis of the skin following subcutaneous injection of complete Freund’s adjuvant (CFA) into the hindpaw. This sprouting correlated with an increase in pain-related sensitivity. We hypothesized that this sprouting and pain-related behavior were caused by an increase in nerve growth factor (NGF) levels. In this study, we investigated whether the inhibition of mature NGF degradation, using a matrix metalloproteinase 2 and 9 (MMP-2/9) inhibitor, was sufficient to reproduce a similar phenotype.

**Results:**

Behavioral tests performed on male Sprague–Dawley rats at 1, 3, 7 and 14 days after intra-plantar MMP-2/9 inhibitor administration demonstrated that acute and chronic injections of the MMP-2/9 inhibitor induced sensitization, in a dose dependent manner, to mechanical, hot and cold stimuli as measured by von Frey filaments, Hargreaves and acetone tests, respectively. Moreover, the protein levels of mature NGF (mNGF) were increased, whereas the levels and enzymatic activity of matrix metalloproteinase 9 were reduced in the glabrous skin of the hind paw. MMP-2/9 inhibition also led to a robust sprouting of sympathetic fibers into the upper dermis but there were no changes in the density of peptidergic nociceptive afferents.

**Conclusions:**

These findings indicate that localized MMP-2/9 inhibition provokes a pattern of sensitization and fiber sprouting comparable to that previously obtained following CFA injection. Accordingly, the modulation of endogenous NGF levels should be considered as a potential therapeutic target for the management of inflammatory pain associated with arthritis.

## Background

Nerve growth factor (NGF) is the prototype of the neurotrophin family of growth factors [1,2]. As a trophic factor, NGF supports the development and survival of peptidergic primary sensory and sympathetic neurons [3]. During early neuronal development, it has a critical role in the survival and growth of sympathetic and sensory neurons [4-6]. Though NGF is no longer required for the survival of sensory neurons in adult animals, a body of evidence has shown that NGF continues to be involved in specifying the function of many sensory neurons and their interactions with sympathetic neurons [7]. Within the mature organism, NGF plays a significant role in mediating inflammatory and immune responses after peripheral tissue injury [8]. NGF levels are elevated in several conditions associated with pain in humans, including arthritis [9,10], cystitis [11,12] and chronic headaches [13]. In animal studies, the concentration of NGF in the skin increases in response to inflammation produced by either injection of irritants [12] or ultraviolet-B irradiation [14]. NGF has been implicated in the sensitization of peripheral nerves to noxious stimuli [15,16].

It has recently been shown by Bruno and Cuello (2006) that NGF is released as a precursor, proNGF, and not in the the growth-promoting form, mature NGF (mNGF) [17]. This work revealed a protease cascade responsible for proNGF maturation into mNGF and its degradation in the extracellular space. One of the key regulatory enzymes involved in proNGF processing within the CNS is plasmin, which is converted from plasminogen by tissue plasminogen activator (tPA) or urokinase plasminogen activator (uPA). In the above report it was proposed that this newly reported metabolic pathway should have an impact on pain mechanisms. However, the validation of this pathway in the periphery and its impact on pain regulation remains unresolved. This biochemical pathway might be particularly relevant for pain because NGF over-expression studies have shown that NGF is an important signal for mechanical and thermal sensitization [16]. Based on the above, we have advanced the hypothesis that interfering with the levels of endogenous mNGF by modulating its formation from proNGF should provide an attractive opportunity to develop a novel class of agents for the treatment of pain. In the present study we aim at exploring whether NGF processing within the periphery is analogous to that already shown in the CNS of naive rats. As a proof of concept, we sought to verify whether the inhibition of endogenous NGF degradation in naïve rats — by the administration of a matrix metalloproteinase 2 and 9 (MMP-2/9) inhibitor — influenced: 1) the protein levels of molecules involved in NGF processing, 2) the innervation patterns of the skin by sensory and autonomic fibers as well as relationship between them, and 3) the pain thresholds.

## Results

### Effect of repeated administration of the MMP-2/9 inhibitor on the protein levels of NGF and MMP-9

**Figure 1:**
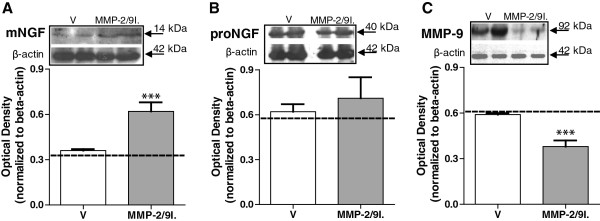
**MMP-2/9 inhibitor administration altered the protein levels of NGF and MMP-9 in the skin of naive rats.** Western blot analysis performed on the glabrous skin samples following 14 days of repeated MMP-2/9 inhibitor (MMP-2/9I.; 20 μg, i.pl.) administration showed a significant increase in the protein levels of mature NGF (mNGF) **(A)**, with no influence on the protein level of the precursor proNGF **(B)**. Repeated administration of MMP-2/9I. (20 μg, i.pl.) significantly reduced the protein levels of MMP-9 **(C)** in the glabrous skin. Examples of representative western blots are presented in the upper panels A, B and C. The densitometry results are presented as the mean ± SEM from all samples. Inter-group differences were analyzed by ANOVA with a Bonferroni’s multiple comparison test. ***p < 0.001 indicates a significant difference as compared to glabrous skin sample of chronic vehicle-treated (V) naive rats. The interrupted line on the graphs indicates protein analyses for mNGF, proNGF or MMP-9 in the contralateral glabrous skin of chronic MMP-2/9I.-treated naïve rats.

Western blot analyses of the glabrous skin samples were carried out on the 14th day after two weeks of chronic injections of the MMP-2/9 inhibitor - 20 μg, intraplantar (i.pl.). This time point and dose for biochemical analyses were selected on the basis of behavioral experiments (described below) where the maximum effect on pain-related behavior was observed on the 14th day of repeated injections of the MMP-2/9 inhibitor at a dose of 20 μg. Both proNGF, mNGF and MMP-9 were identified based on their molecular weights; proNGF migrated close to 40 kDa, mNGF migrated at 14 kDa, and MMP-9 migrated at 92 kDa. The localization of these bands is consistent with what is described in other publications using the same antibodies [17-19]. Furthermore, they aligned with the corresponding bands from positive controls (mouse submandibular gland extracts for NGF and kidney homogenates for MMP-9) (data not shown). Our data revealed an increase in the protein levels of the mature form of NGF (mNGF) in the ipsilateral paw as compared to the contralateral paw (shown as an interrupted line on the graphs, 0.62 ± 0.06 vs 0.33 ± 0.02; Figure 1A) and also when compared to ipsilateral paw samples from vehicle-treated rats (0.62 ± 0.06 vs 0.36 ± 0.01). No significant changes in the protein levels of the precursor form of NGF (proNGF) were detected by Western blot analysis in the ipsilateral paw as compared to the results from the contralateral paw (0.71 ± 0.14 vs 0.59 ± 0.03; Figure 1B) and to the ipsilateral paw of vehicle-treated rats (0.71 ± 0.14 vs 0.62 ± 0.05). In addition, the treatment with the MMP-2/9 inhibitor resulted in the decrease of MMP-9 protein levels in the ipsilateral paw when compared to the contralateral side (0.38 ± 0.04 vs 0.61 ± 0.03; Figure 1C) and when compared to the ipsilateral paw of vehicle-treated rats (0.38 ± 0.04 vs 0.62 ± 0.01). To validate this observation, we pre-treated the samples with urea, a strong denaturing agent that promotes protein unfolding [20]. This treatment ensured that the primary antibody against MMP-9 was in fact recognizing its antigenic site and that the binding was not being masked by the MMP-2/9 inhibitor binding. Indeed, we did not find any difference in the data after urea pre-treatment, an observation that provides sufficient evidence that the protein level is in fact decreased (data not shown).

### Effect of repeated administration of the MMP-2/9 inhibitor on the enzymatic activity of MMP-9

**Figure 2:**
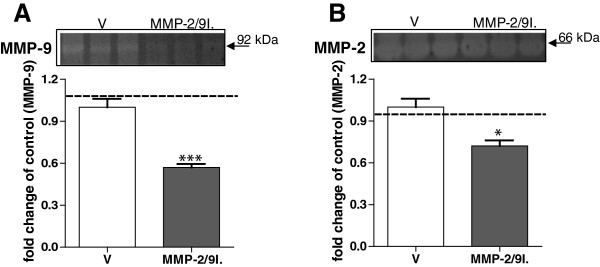
**Effect of MMP-2/9 inhibition on the enzymatic activity of MMP-9 and MMP-2 in the skin of naive rats.** To evaluate MMP activity following repeated MMP-2/9 inhibition (MMP-2/9I.; 20 μg, i.pl.), zymography was performed on extracts from glabrous skin samples. The analysis revealed reduction in the enzymatic activity of both MMP-9 **(A)** and MMP-2 **(B)**. Examples of representative zymograms are presented in the upper panels A and B. The densitometry results are presented as the mean ± SEM. Inter-group differences were analyzed by ANOVA with a Bonferroni’s multiple comparison test. *p < 0.05, ***p < 0.001 indicate a significant difference as compared to glabrous skin sample of vehicle-treated (V) rats. The interrupted line on the graphs indicates zymogram analyses for MMP-9 and MMP-2 in the contralateral glabrous skin of chronic MMP-2/9I.-treated naïve rats.

Zymography analyses performed on the glabrous skin samples from the hind paws revealed changes in the enzymatic activity of both MMP-9 (82-kDa) and MMP-2 (65-kDa) following the daily subcutaneous administration of MMP-2/9 inhibitor (20 μg, i.pl.; Figure 2A, B). The treatment with the MMP-2/9 inhibitor reduced the enzymatic activity of MMP-9 and MMP-2 in the ipsilateral paw when compared to the contralateral paw (shown as an interrupted line on the graphs, 0.57 ± 0.03 vs 1.14 ± 0.05 and 0.72 ± 0.04 vs 0.94 ± 0.06, respectively) and when compared to ipsilateral skin samples from vehicle-treated rats (0.57 ± 0.03 vs 1.0 ± 0.06 and 0.72 ± 0.04 vs 1.0 ± 0.06, respectively).

### Effect of the repeated administration of MMP-2/9 inhibitor on the skin innervation pattern

**Figure 3:**
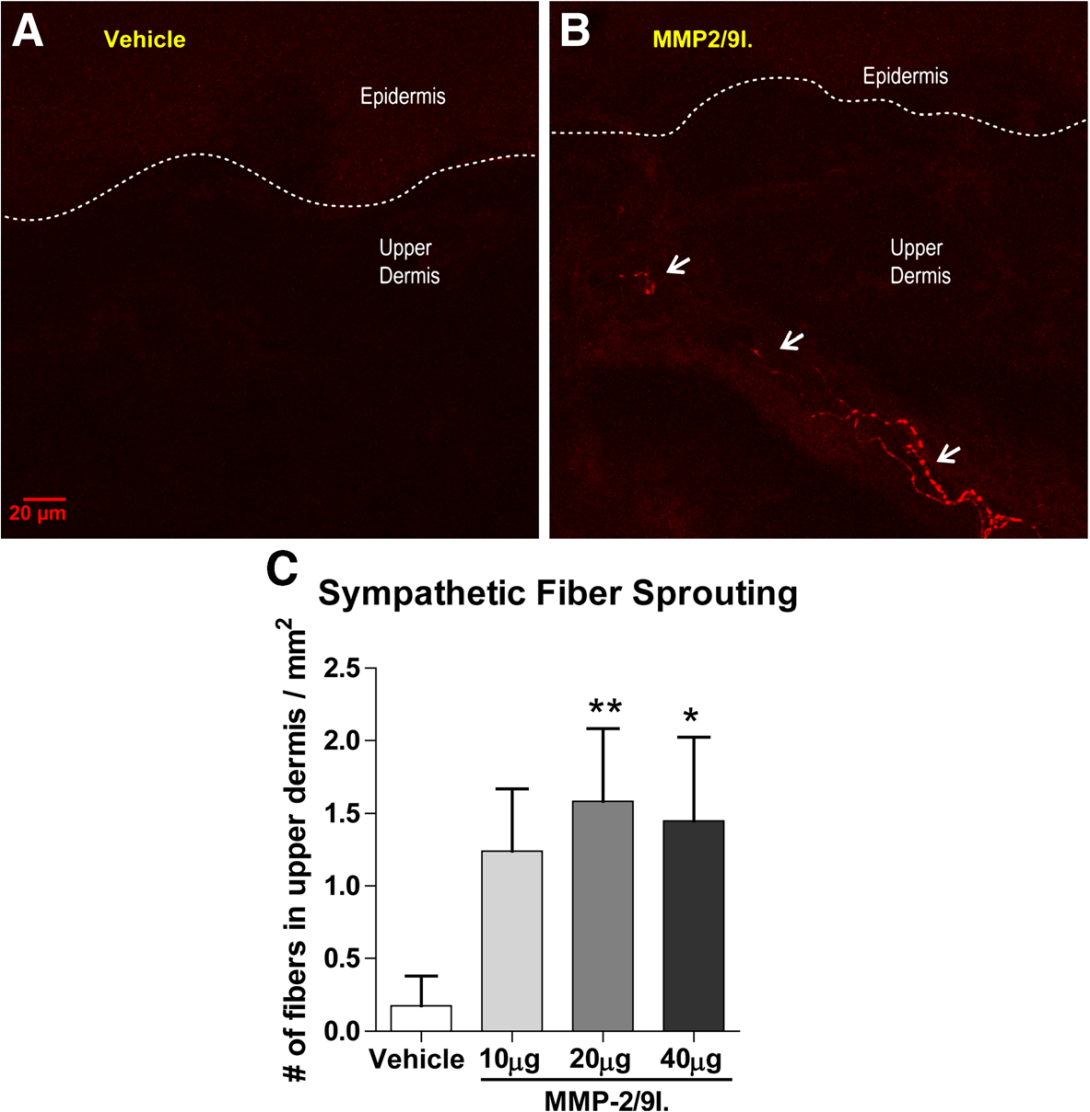
**Changes in sympathetic fiber innervation in the skin of naïve rats following MMP-2/9 inhibition. A** and **B** represent confocal images of the rat’s glabrous hindpaw skin in vehicle-treated and MMP-2/9 inhibitor (MMP-2/9I.; 20 μg, i.pl.) injected rats, respectively, at the 2 week end point. Note an invasion of sympathetic fibers into the upper dermis **(B)**, mimicking what we had previously observed in a model of inflammatory arthritis [35]. This fiber population was not normally found in control (vehicle-treated) animals as shown in A. Arrows in B indicate VMAT2-IR fibers in the upper dermis. The results in **C** are from all experimental groups and presented as the mean ± SEM. Inter-group differences were analyzed by ANOVA with a Dunnett’s post hoc test. *p < 0.05, **p < 0.01 indicates a significant difference as compared to glabrous skin sample of chronic vehicle-treated (V) naive rats. The dashed lines represent the dermal-epidermal junction.

**Figure 4:**
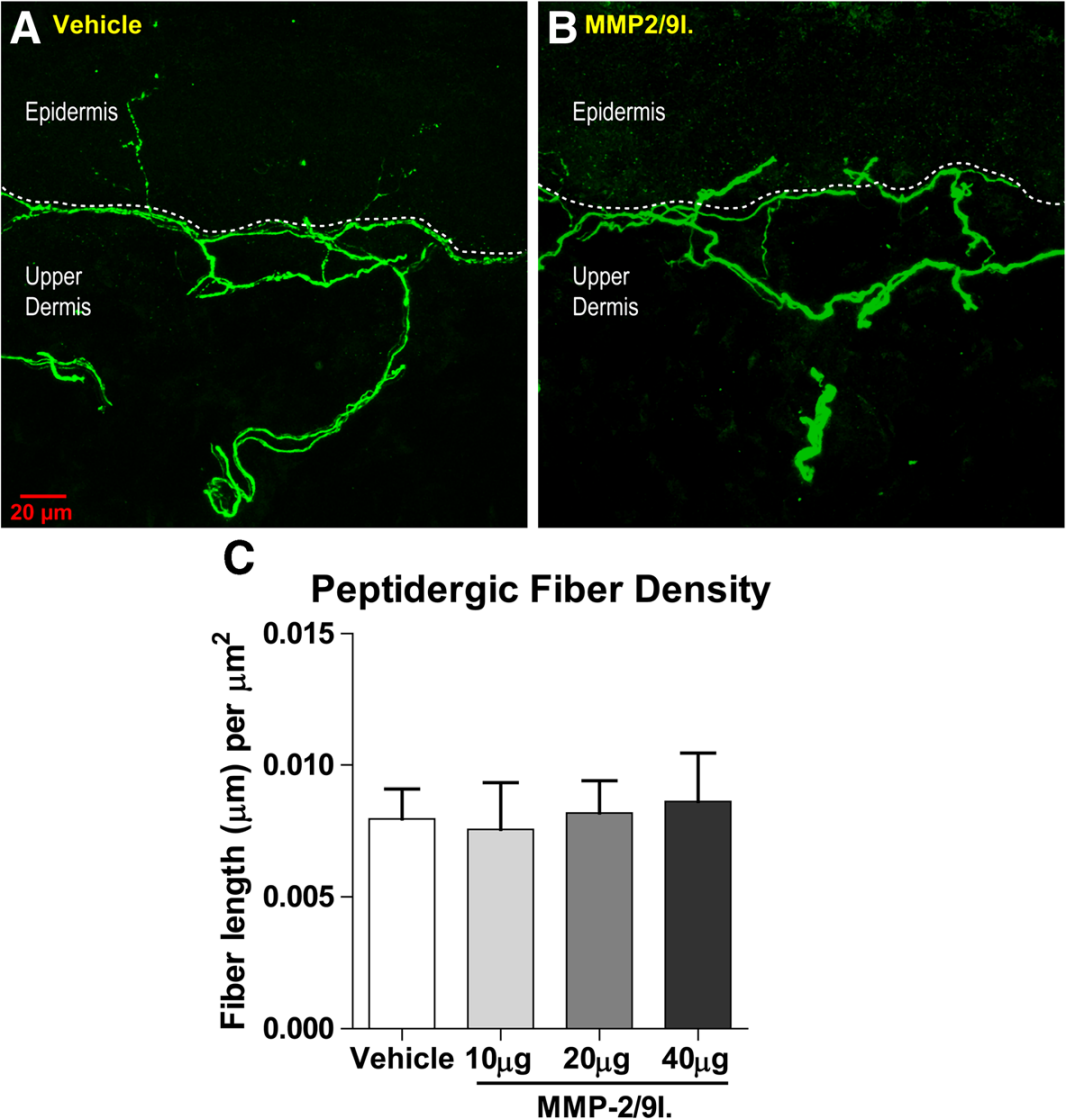
**Density of sensory fibers in the skin of naïve rats following matrix MMP-2/9 inhibition.** The density of CGRP-positive fibers following repeated matrix MMP-2/9 inhibitor (MMP-2/9I.; 10, 20, 40 μg, i.pl.) administration did not change significantly from control levels **(C)**. **A** and **B** are representative confocal images of the rat’s glabrous hindpaw skin in vehicle-treated and MMP-2/9 inhibitor (MMP-2/9I.; 20 μg, i.pl.) injected rats, respectively. The results are presented as the mean ± SEM. Inter-group differences were analyzed by ANOVA with a Dunnett’s post hoc test. The dashed line represents the dermal-epidermal junction.

**Figure 5:**
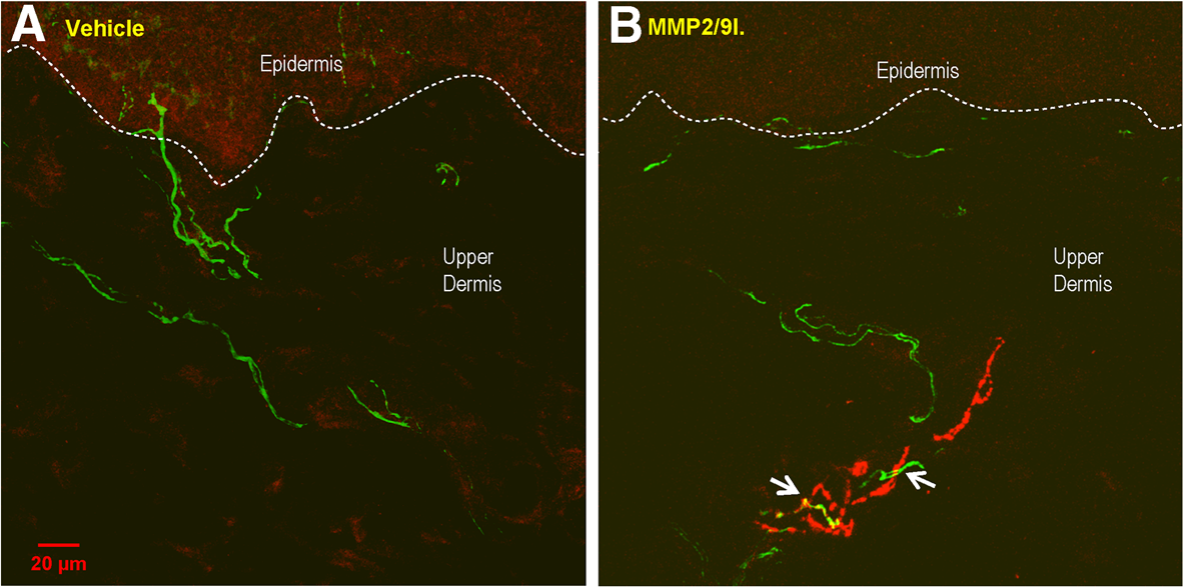
**Sympathetic/sensory fiber interaction in the skin of naïve rats following MMP-2/9 inhibition.** Note that close appositions of ectopic sympathetic fibers (VMAT2-IR) with peptidergic nociceptive fibers (CGRP-IR) in the upper dermis were observed at the 2 week end point following repeated injections of MMP-2/9 inhibitor (MMP-2/9I.; 20 μg, i.pl.). **A** and **B** represent confocal images of the rat’s glabrous hindpaw skin in vehicle-treated and MMP-2/9 inhibitor (MMP-2/9I.; 20 μg, i.pl.) injected rats, respectively.

In the current study, we hypothesized that inhibition of MMP-9 in naïve animals would prevent the degradation of endogenous mNGF and lead to sympathetic fiber sprouting in the skin. Indeed, there was an invasion of the upper dermis by sympathetic fibers, as detected by VMAT-2 immunoreactivity, in skin samples from animals treated for 2 weeks with daily injections of MMP-2/9 inhibitor (Figure 3). The increased number of sympathetic fibers in the scanned area of the upper dermis was statistically significant at doses of 20 and 40 μg of MMP-2/9 inhibitor. Interestingly, the density of peptidergic sensory fibers, as detected by CGRP immunoreactivity, did not change compared to control levels (Figure 4). One interesting observation in MMP-2/9 inhibitor treated animals was that, in the upper dermis, the VMAT-2-immunoreactive fibers were often observed wrapping around CGRP-IR fibers (Figure 5).

### Effect of a single administration of the MMP-2/9 inhibitor on allodynia and hyperalgesia

**Figure 6:**
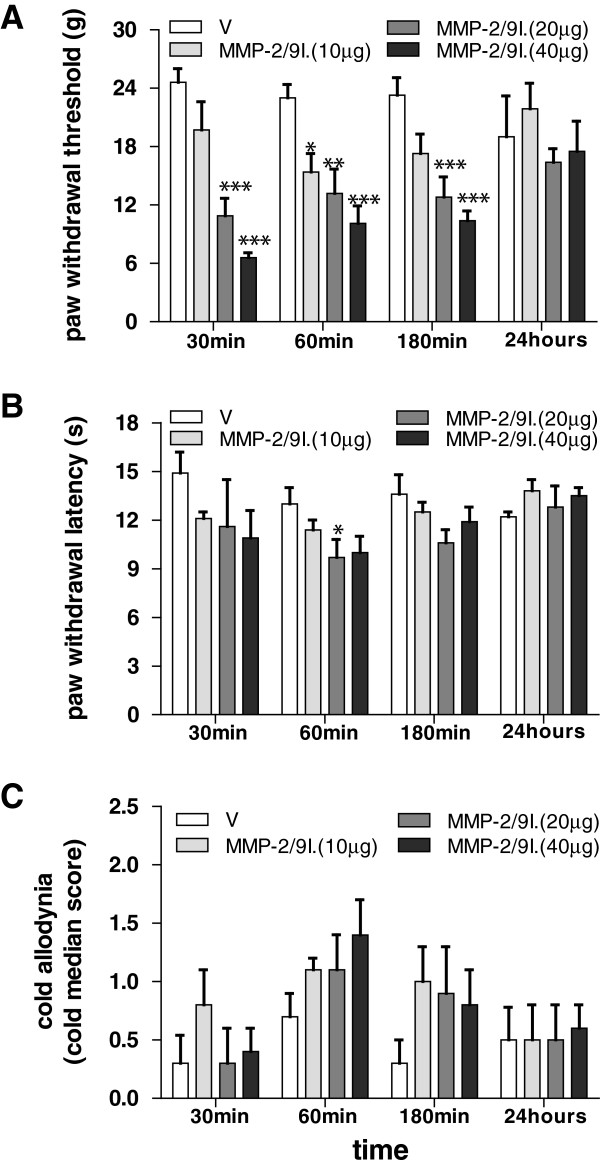
**Effect of a single MMP-2/9 inhibitor administration on allodynia and hyperalgesia in naive rats.** A single intra-plantar administration of MMP-2/9 inhibitor (MMP-2/9I.; 10, 20, 40 μg, i.pl.) induced mechanical allodynia and thermal hyperalgesia as measured by von Frey and Hargreaves tests. However, the treatment had no effect on cold allodynia as measured by acetone test. Behavior: the von Frey **(A)**, Hargreaves **(B)** and acetone **(C)** tests were performed 30, 60, 180 min and 24 h after MMP-2/9 inhibitor administration. Results are presented as the mean ± SEM (n = 6–8 rats/group). Inter-group differences were analyzed by Bonferroni’s multiple comparison test. *p < 0.05, **p < 0.01, ***p < 0.001, compared to vehicle-treated (V) naïve rats.

A single subcutaneous i.pl. administration of matrix MMP-2/9 inhibitor (10, 20, 40 μg) induced mechanical allodynia in naive rats, as measured by the von Frey test. Pain-related behavior was evaluated at 30, 60, 180 minutes and 24 hours after single injection of this inhibitor (Figure 6A). The MMP-2/9 inhibitor (20 μg; i.pl.) induced thermal hyperalgesia as measured by the Hargreaves test at 60 minutes following a single injection (Figure 6B). In contrast, a single injection of the MMP-2/9 inhibitor (10, 20, 40 μg) did not influence cold allodynia as assessed by the acetone test at 30, 60, 180 minutes and 24 hours after the injection (Figure 6C).

### Effect of repeated administration of the MMP-2/9 inhibitor on allodynia and hyperalgesia

**Figure 7:**
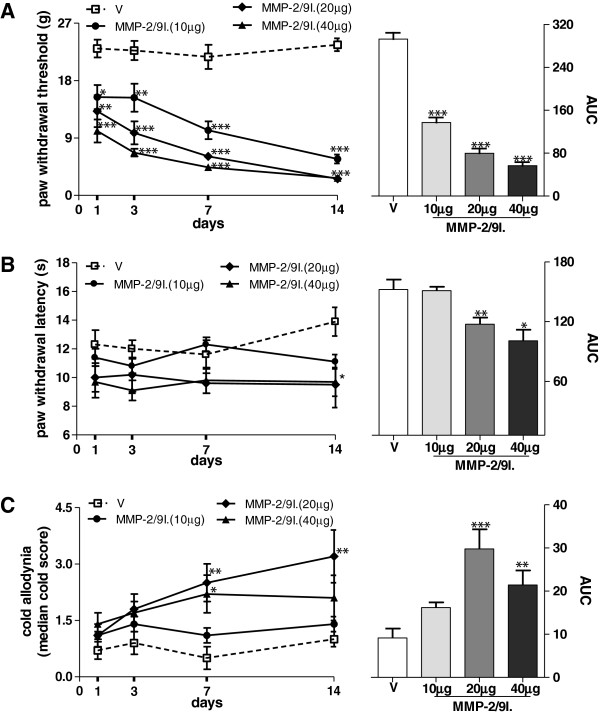
**Effect of repeated MMP-2/9 inhibition on allodynia and hyperalgesia in naive rats.** Repeated intra-plantar administration of MMP-2/9 inhibitor (MMP-2/9I.; 10, 20, 40 μg, i.pl.) once daily for fourteen days induced allodynia and hyperalgesia. Behavioral tests: the von Frey test **(A)**, Hargreaves test **(B)** and acetone test **(C)** were performed on the 1st, 3rd, 7th and 14th day of repeated MMP-2/9 inhibitor injections. Results are presented as the mean ± SEM (n = 6–8 rats/group). Inter-group differences were analyzed by Bonferroni’s multiple comparison test. *p < 0.05, **p < 0.01, ***p < 0.001, compared to vehicle-treated (V) naïve rats. The area under the curve (AUC) was calculated by the Trapezoidal & Simpson’s Rules, Pharmacologic Calculation System, version 4.0-03/11/86. Inter-group differences were analyzed by Bonferroni’s Multiple Comparison Test. *p < 0.05, **p < 0.01, ***p < 0.001 indicate a significant difference as compared to chronic vehicle-treated (V) naive rats.

The repeated administration of MMP-2/9 inhibitor (10, 20, 40 μg; i.pl.) to naïve rats once a day for two weeks resulted in the development of hypersensitivity to both mechanical and thermal stimuli as measured by the von Frey, Hargreaves and acetone tests, respectively (Figure 7). The sensitivity to both mechanical and thermal stimuli increased over time. The analysis of the area under the curve (AUC), which allows for the assessment of the global effect of treatment, shows that the MMP-2/9 inhibitor treatment induced a significant allodynia and hyperalgesia in naïve rats (p < 0.05) (Figure 7A,B,C; right panels). We did not observe any visible signs of inflammation (reddening or swelling of the skin) throughout the 14 days period at the MMP-2/9 inhibitor or vehicle injection site. Moreover, we found a statistically significant positive correlation between the number of sympathetic fibers in the upper dermis of the skin and mechanical allodynia, as measured by the von Frey test (p = 0.0029), and with cold allodynia, as measured with the acetone test (p = 0.0086). There was no positive correlation between the thermal hyperalgesia measured by the Hargreaves test and the sympathetic sprouting, although there was trend (p = 0.08).

## Discussion

The present study provides evidence that the intra-plantar administration of an MMP-2/9 inhibitor induced sensitization to mechanical, heat and cold stimuli in naïve rats. Our data also shows that the protein levels of mNGF and MMP-9 were affected in the hind paw skin following repeated MMP-2/9 inhibitor injections. Furthermore, we found that the MMP-2/9 inhibitor induced sprouting of sympathetic fibers into the upper dermis in the glabrous hind paw skin, but no change in the density of sensory peptidergic fibers. Our results support the hypothesis that sympathetic sprouting and pain related-behavior are due to an increase in mNGF levels.

### NGF metabolic pathway

NGF is secreted from cells in an activity dependent manner as a precursor (proNGF), together with a full enzymatic cascade capable of leading to its breakdown into the mature (active) form and its degradation [17]. The existence of the CNS protease cascade responsible for the conversion of proNGF into mNGF and for its degradation was demonstrated by a series of *ex vivo* as well as *in vivo* studies [17] that defined the relevance of tPA/plasminogen activation in the proNGF to mNGF conversion and of MMP-9 in the enzymatic degradation of mNGF. Although this NGF metabolic pathway is now well established in the CNS [18,21], it has been much less studied in the periphery. The significance of such studies arise from the fact that up-regulation of NGF is a cause of many clinical conditions associated with pain [9-12,22-25]. Therefore, understanding the mechanism underlying NGF processing and its eventual modulation provides attractive therapeutic opportunities. Our results provide for the first time an important validation in the periphery of the role of MMP-9 in the mNGF degradation. Indeed, we were able to show that repeated intra-plantar administration of an MMP-2/9 inhibitor resulted in elevated levels of mNGF in the hind paw skin. Interfering with the degradation of endogenous mNGF was also sufficient to induce sprouting of sympathetic fibers in the skin and hypersensitivity to both mechanical and thermal stimulation. Our results confirm and expand the hypothesis that NGF plays an important role in pain mechanisms (see below).

### NGF induced fiber sprouting

NGF is a crucial neurotrophin for the definition of the innervation pattern of the skin during development [26]. Adult sympathetic fibers are regulated by NGF in what regards their survival and phenotypic maintenance [27,28] and can respond with fiber outgrowth to supplemental NGF [29-32]. In the present study, we were able to show that inhibition of MMP-9 leads to an increase in mNGF levels and, likely as a consequence, to pain-related behaviors and a sprouting of sympathetic fibers into the upper dermis, a region of skin which is normally devoid of this fiber population. Interestingly, we did not observe any changes in the density of peptidergic sensory fibers following MMP-2/9 inhibitor injections. It has been demonstrated that sympathetic fibers are more responsive to NGF in the adult as they depend on it for survival, although NGF still plays a role in maintaining the phenotype of sensory peptidergic neurons [33,34]. Therefore, the lack of peptidergic sprouting in the current study would suggest that the levels of mNGF in the skin resulted from MMP-2/9 inhibition were lower than in the chronic inflammation model [35]. This is to be expected, because following a local subcutaneous injection of CFA there is extensive and long lasting skin inflammation which likely leads to sustained elevated local levels of mNGF. In the present study, we demonstrate that preventing the degradation of the NGF in the absence of any pathology is sufficient to increase the levels of endogenous mNGF. We have also observed that, in such conditions, the sympathetic fibers sprouted in a pattern comparable to what we previously observed in the arthritis model [35] and also in neuropathic pain models [36,37]. These studies demonstrate that the ectopic sympathetic fibers are not associated with blood vessels and often wrap around sensory peptidergic fibers suggesting enhanced sympathetic-sensory interaction. Under normal physiological conditions, the sympathetic nervous system is not associated with nociception and uninjured primary sensory nerve endings are not sensitive to catecholamines. However, noradrenaline injection following nerve lesions has been shown to excite small diameter nociceptors, but not in normal conditions [38,39]. This is in agreement with the sympathetic participation in some forms of neuropathic pain [40]. One possible mechanism for the role of the sympathetic system in pathological pain is the ectopic association of sympathetic fibers with sensory peptidergic fibers in upper dermis of the skin [37,41]. Curiously, a similar ectopic association of sensory and sympathetic fibers was also observed in the thick skin of the rat hind paw following chronic inflammation [35]. We observed here a similar fiber remodeling which paralleled the pain-like behavior following MMP-9 inhibition, in the absence of any lesion. Additionally, our results concur with previous studies showing that sympathetic sprouting is related to increased levels of NGF. Thus, it has been shown that intraventricular infusion of nerve growth factor *per se* is able to induce sympathetic fiber sprouting in the sensory ganglia [42], subpial region of the medulla, spinal cord [43] and in the intracranial vasculature [44,45] of naïve rats. Conversely, surgical or chemical sympathectomy has been shown to reduce the thermal and mechanical hyperalgesia evoked by NGF [9,46,47].

### NGF-evoked hyperalgesia

It has been shown that exogenous treatment with NGF evoked nociceptive behavior in animals [46,48-51] and induced pain in humans [52,53]. A single NGF injection into the skin induced long-lasting mechanical and thermal hypersensitivity in humans [54,55]. Also, recently published data obtained in the rodent reported that the intra-plantar injection of NGF induced an immediate, long-lasting increase in mechanical and thermal sensitivity, as early as one hour post-injection [56]. This study reported that the effect on thermal sensitivity was of shorter duration compared with that on mechanical sensitivity, what is in agreement with our results and indicates that mechanical hypersensitivity requires a considerable longer time to resolve than the heat hypersensitivity. This situation is similar to that in the human, where heat hypersensitivity resolved much faster than the long-lasting increase in mechanical sensitivity [56]. In the current study, we observed changes in pain-related behavior when we inhibited NGF degradation in naïve rats, with a different time course for mechanical and thermal sensitivities. The temporal differences in resolution between mechanical and thermal sensitivity suggest different mechanisms, however what drives these differences is still unclear. One of the possible mechanisms involved in NGF-induced hyperalgesia might be through sympathetic sprouting and abnormal sympathetic-sensory interactions [57,58]. Indeed, sympathetic fibers express trkA receptors and their activation results in noradrenaline release which would increase in excitability and spontaneous activity of petidergic sensory neurons [57,58]. Also ATP, a co-transmitter released from sympathetic fibers and damaged tissues [59,60], has been shown to play a role in activating primary afferents via purinergic receptors [61] and may play a role in the hyperexcitability as well. Recent data from our laboratory provided some support to these sympathetic-sensory interactions in an inflammatory arthritis animal model, in which there is sympathetic fiber sprouting in skin over the joints as well as in the arthritic joint. Indeed, in that study we have shown that pharmacological suppression of sympathetic fiber function with systemic guanethidine significantly decreased the pain-related behavior associated with inflammatory arthritis [62]. Our data following guanethidine administration was comparable with that obtained in other chronic pain models [63,64]. Moreover, there is also evidence from the literature that the sodium channel Nav 1.8, expressed on sensory neurons, may be a major player in NGF-induced thermal hyperalgesia [65]. Evidence shows that NGF modulates this sodium channel current density through the PKA pathway following trkA binding [66]. These changes in sodium channels might occur even in the absence of peptidergic fibre sprouting as consequence of mNGF increases induced by MMP-2/9 inhibition. Since many NGF-responsive neurons contain the vanilloid receptor TRPV1, this receptor is also suspected to play a role in NGF-mediated hypersensitivity [67-69]. Cultured DRG neurons treated with NGF display enhanced inward currents in response to the application of the TRPV1 agonist capsaicin [70,71]. NGF can increase TRPV1 expression [72,73] and promote TRPV1 insertion into the plasma membrane [74]. NGF also acts indirectly by activating mast cells and neutrophils, which in turn release additional inflammatory mediators causing hypersensitivity [46,47,75-77]. There is a growing body of evidence indicating that NGF-evoked hyperalgesia is caused by a sensitization of primary afferent nociceptors that supply an area of damage or inflammation [78,79].

Matrix metalloproteinases have multiple functions. Therefore, it is not surprising that there is evidence that MMP-9 inhibition at the spinal cord level alleviates the early phase of neuropathic pain, whereas intrathecal administration of MMP-9 itself is sufficient to produce neuropathic pain-like symptoms in normal animals [80]. These results at the spinal cord level are very different from those we obtained through a peripheral inhibition of MMP-9, likely because, in the CNS, the MMP-9-induced pathophysiology involves interleukin-1β cleavage to its active form and microglial p38 activation [80], rather than effects through NGF. However, because MMP-2 is also responsible for the degradation of several pain-related mediators such as TNF-α, interleukin-1β, and substance P [81], we cannot rule out that MMP-2 inhibition might increase the levels of these mediators and subsequently contribute to the hyperalgesia through a peripheral mechanim.

## Conclusions

Our data provides evidence that the metabolic processing of endogenous NGF can be modulated in the periphery in a manner similar to what was previously demonstrated in the central nervous system. We show that inhibition of NGF degradation in naïve rats induces sprouting of sympathetic fibers into the upper dermis of the skin, where they wrap around the peptidergic nociceptive afferents, suggesting an interaction between these two fiber populations. We propose that the observed sensitization to both mechanical and thermal stimuli is a consequence of elevated endogenous mNGF levels sensitizing peptidergic primary afferents and promoting abnormal sensory-autonomic fiber interactions in the skin. As NGF has been strongly implicated in different pain conditions, modulating its metabolic pathway provides an opportunity to influence its endogenous levels to alleviate pain.

## Methods

### Animals

Adult male Sprague–Dawley rats (275–300 g) were used in this study. Animals were housed in groups of four per cage with sawdust bedding under a standard 12 h/12 h light/dark cycle (lights on at 08.00 AM) with food and water available *ad libitum*. All experiments were carried out according to protocols approved by the McGill University Animal Care Committee and followed the guidelines for animal research from the International Association for the Study of Pain (IASP) [82].

### Drug administration

An MMP-2/9 inhibitor (MMP-2/MMP-9 Inhibitor I, EMD Chemicals, Inc.), which selectively inhibits both MMP-2 (IC50 = 310 nM) and MMP-9 (IC50 = 240 nM), was administered subcutaneously into the plantar region of the right hind paw, once a day, for 14 days. The control group of rats received vehicle (DMSO, 20 mg/1 ml) according to the same schedule. The doses of MMP-2/9 inhibitor were selected based on a previous study from our lab [18] and our preliminary experiments. The behavioral tests were conducted 30, 60, 180 minutes and 24 hours after the first MMP-2/9 inhibitor injection and then 60 min following the MMP-2/9 administration on days 3, 7 and 14.

### Behavioral tests

Signs of mechanical, heat and cold hypersensitivity were detected using the von Frey, Hargreaves and acetone tests, respectively. Prior to any behavioral testing, animals were habituated three times to the testing environment. The baseline reaction values were measured one day before the first MMP-2/9 injection and were as follows: 26 g for the von Frey test, 12.2 ± 1.9 s for the Hargreaves test and 0.5 ± 0.2 for acetone test.

### Mechanical allodynia (von Frey test)

Mechanical allodynia in rats was measured using a series of calibrated nylon von Frey filaments (Stoelting, Wood Dale, IL, USA), ranging from 0.6 to 26 g. Animals were placed in plastic cages with a wire-mesh floor. They were placed in this environment approximately 5 min before testing in order to allow behavioral accommodation. The von Frey filaments were applied in ascending order to the midplantar surface of the injured hindpaw through the mesh floor. Each probe was applied to the foot until the filament bent. The time interval between consecutive filament administrations was at least 5 s. The calculations were performed as described previously [83].

### Thermal hyperalgesia (Hargreaves’ test)

The pain threshold to high temperature was tested using the Plantar Test (Hargreaves Apparatus, Ugo Basile, Type 7370, Comerio, Varese, Italy). Rats were placed into individual plastic cages with glass floors 5 min before the experiment. A noxious thermal stimulus was focused through the glass onto the plantar surface of a hind paw until the animal lifted the paw away from the heat source. The paw withdrawal latency was automatically measured to the nearest 0.1 s. A cut-off latency of 20 s was used to avoid tissue damage. The latency to the nociceptive reaction was measured in seconds under basal condition and after drug treatment.

### Thermal allodynia (acetone test)

Cold allodynia was assessed using the acetone drop method [84]. In the test environment described above, a 50 μl droplet of acetone was applied to the midplantar hind paw using a micropipette. Responses within the first 20 s were scored according to the following rating system: 0 - no response; 1- one rapid hind paw flick/stamp; 2 - two or more hind paw flicks/stamps; 3 - periods of flicking/stamping with licking of plantar hind paw [85]. The acetone application was repeated three times for each hind paw, with a 3 min interval between each application. For each rat, the sum of the three scores was used for data analysis.

### Biochemistry

#### Western blot

Following fourteen days of daily MMP-2/9 inhibitor administration, the rats were decapitated and the glabrous skin from the hind paws was collected, frozen in the liquid nitrogen and kept for further processing. Tissue samples were homogenized in RIPA buffer (1% NP-40, 1% sodium deoxycholate, 0.1% sodium dodecyl sulfate, 150 mM NaCl, 25 mL Tris–HCl, pH 7.6) containing protease inhibitors (Complete, Roche Molecular Biochemicals, Indianapolis, IN) and cleared by centrifugation (13,000 rpm for 50 min at 4°C). Protein concentration of the supernatant was determined using the BCA Protein Assay Kit (Sigma). Homogenates (20 μL of 50 μg of total protein) were resolved in 4-12% polyacrylamide gels and transferred into a nitrocellulose membrane (Bio-Rad Laboratories, Inc). The blots were blocked in Tris Buffer Solution-Tween 20 (TBS-T) containing 5% non-fat powdered milk at room temperature for 1 h on a rotomixer. Nitrocellulose membranes were probed with primary antibodies specific for nerve growth factor (NGF, 1:500, E2610, Santa Cruz Biotechnology) and matrix metalloproteinase-9 (MMP-9, 1:500, AB19016, Millipore). Blots were incubated overnight at 4°C with the primary antibodies. After primary antibody incubation, membranes were washed 3 × 10 min in TBS-T. This was followed by incubation for 2 h at room temperature with a peroxidase-conjugated goat anti-rabbit IgG antibody (1:2500) (Jackson Immuno Research Laboratories, Inc.). The membranes were washed 3 × 10 min in TBS-T. The immunoreactive bands were visualized with the ECL enhanced chemiluminescence kit (PerkinElmer Inc.) and using Kodak Biomax XAR imaging film. The immunoreactive bands were quantified by densitometry of the films using a MCID M4 image analysis system (Imaging Research Inc., St. Catharines, ON, Canada). Membranes were rinsed and reprobed with a mouse anti-β-actin antibody (1:40,000; Sigma) diluted in 5% milk in TBS-T for 1 h at room temperature, washed with TBS-T, and incubated with a peroxidase conjugated donkey anti-mouse IgG (1:5000, Santa Cruz) in 5% dry milk in TBS-T for 1 h. The membranes were washed and the signal was detected and qualified as described above. The levels of NGF and MMP-9 were normalized to the β-actin levels for each sample.

#### Zymography

Gelatinolytic activity was determined by zymography using gelatin-containing gels following the protocol provided by supplier Millipore. The skin samples were collected at the same time point as those for western blot analysis (see above). The skin sample extracts were mixed with an equal volume of non-reducing sample buffer (0.5 M Tris–HCl, pH 6.8, SDS, glycerol and bromophenol blue). The samples (50 μg of protein) were electrophoresed on an 8% SDS–PAGE containing 0.1% gelatin as the substrate. After electrophoretic separation, the gels were incubated for 30 min in renaturing buffer (2.5% Triton X-100 in distilled water) to remove the SDS, washed for 2X10 min with water and then incubated for 24 h at 37°C in a developing buffer containing 50 mM Tris–HCl, pH 7.78; 5 mM CaCl2 and 0.02% Brij 35. Following incubation, the gels were stained for 1 h with 0.5% Coomassie R-250 staining solution and then differentiated in a solution of methanol: acetic acid: water (50 : 10 : 40). Enzyme activity attributed to MMP-2 and MMP-9 was visualized (on the basis of molecular weight) in the gelatin-containing zymograms as clear bands against a blue background. The bands were quantified by densitometry using the MCID M4 image analysis system.

### Immunohistochemistry

Two weeks after the daily injections of MMP-2/9 inhibitor, animals were deeply anesthetized with Equithesin (6.5 mg chloral hydrate and 3 mg sodium pentobarbital in a volume of 0.3 mL, i.p., per 100 g body weight) and then perfused through the left cardiac ventricle with 100 mL of perfusion buffer, followed by 500 mL of 4% paraformaldehyde (PFA) in 0.1 M phosphate-buffer (PB), pH 7.4, at room temperature for 30 minutes. Subsequently, the plantar glabrous skin was extracted and post-fixed in the same fixative for 1 hour at 4°C. The tissue was cryoprotected in 30% sucrose in PB overnight at 4°C for later immunohistochemical processing. Fifty-μm thick cross sections of skin were cut using a cryostat (Leica, Wetzlar, Germany). All sections were collected as free-floating in phosphate-buffered saline (PBS) with 0.2% Triton-X 100 (PBS-T). The tissue sections were incubated for 1 hour at room temperature in 10% normal goat serum (Gibco, Carlsbad, CA) in PBS to block unspecific labeling. To label the peptidergic and sympathetic fiber populations, the sections were then incubated at 4°C for 24 hours using either a rabbit anti-Calcitonin Gene Related Protein (CGRP) (Sigma-Aldrich, St. Louis, MO, C-8198, lot# 070 M4835, diluted 1:2000) antibody or a rabbit anti-Vesicular Monoamine Transporter-2 (VMAT-2) (Phoenix Pharmaceuticals, Inc., CA, USA, H-V004, 01237–1, diluted 1:7500). As controls, some sections were processed omitting the primary antibody; no specific staining was observed. After three rinses in PBS-T, the sections were incubated for 2 hours at room temperature with goat anti-rabbit IgG conjugated to either Alexa Fluor 488 for CGRP or Alexa Fluor 594 for VMAT-2 (Molecular Probes, diluted 1:800). For double-labeling of CGRP and VMAT2, sections were processed as described above except that we used a guinea pig anti-CGRP antibody at a 1:4000 dilution (Peninsula, San Carlos, CA, T-5053) in a simultaneous incubation with the rabbit anti-VMAT2 antibody; after washing, we used a mixture of an anti-guinea pig IgG antibody conjugated to Alexa Fluor 488 (1:800; Molecular Probes) with the anti-rabbit IgG conjugated to Alexa Fluor 594. Finally, the sections were washed, mounted on gelatin-subbed slides, air-dried and cover slipped with an anti-fading mounting medium (Aqua PolyMount, Polysciences Inc., Warrington, Pa.). Slides were stored at 4°C until examined. Since we aimed to localize and evaluate the morphological changes in innervation and to quantify the relative changes in the immunostaining intensity, the conditions of all procedures (dilutions of reagents and antibodies, washings, incubation time and temperature, blocking of nonspecific staining), were kept rigorously throughout the assays and were identical for the sections from all tested groups. Within each experiment, immunohistochemical processing of tissue sections sampled from all groups was carried out simultaneously. Before quantitative analyses, all the slides were coded so that the person who performed the quantification was completely blinded regarding the experimental groups. Codes were broken only after the quantification was completed.

#### CGRP-immunoreactive fiber quantification

Quantitative analyses were performed on sections of glabrous skin from the hindpaw ipsilaterally to the injection of MMP-2/9 inhibitor or vehicle. In this study, as in previous work from our laboratory, we have divided the dermis into upper and lower dermis. We have defined the upper dermis as the area of the dermis spanning 150 μm below the dermal-epidermal junction [37]. For the measurement of the density of peptidergic fibers, as detected by CGRP immunoreactivity, we used a Zeiss Axioplan 2 imaging fluorescence microscope equipped with a PlanFluotar 40X oil-immersion objective. This microscope has a high resolution digital camera connected to a computer equipped with the Zeiss Axiovision 4.8 software (Carl Zeiss, Canada). Three sections per slide were randomly selected, and 6 microscopic fields including upper dermis were photographed at random, for a total of 18 images per animal. Images were exported in the TIFF format for analysis with an MCID Elite image analysis system (Imaging Research Inc., St. Catharines, ON, Canada). The upper dermis was outlined with the use of a tracing tool in the software. CGRP-IR fibers were automatically detected by the software using a brightness threshold and converted to 1 pixel in thickness to compute the total fiber length (μm) per scan area (μm^2^).

#### Sympathetic fiber quantification

We used a different approach to quantify the changes in autonomic innervation. Since these fibers are much less abundant in the skin than the sensory, especially in the upper dermis, it was unpractical to measure fiber density. Therefore, we proceeded by photographing 6 entire skin sections per animal and counting all VMAT-2-IR fibers within the upper dermis, measured to be 150 μm from the dermal-epidermal junction. The mean number of fibers in the upper dermis per total area (μm^2^) was then calculated.

### Data analyses

The Western blot and zymography results represent the densitometry analyses of all samples (4–6 samples per group). Inter-group differences were analyzed by one-way ANOVA and Bonferroni’s multiple comparison tests (Figures 1 and 2). The mean number of sympathetic fibers and density of sensory peptidergic fibers in the upper dermis was compared between groups by a one-way ANOVA and a Dunnett’s post hoc test, with a statistical significance accepted at p <0.05 (Figures 3, 4 and 5). As no significant difference was detected among the control groups of all time points, they were pooled. The behavioral data are presented as the mean ± SEM (6–8 animals per group). The effect of single as well as repeated intra-plantar injections of MMP-2/9 inhibitor on mechanical and thermal sensitivity in rats (Figures 6 and 7) was analyzed by one-way analysis of variance (ANOVA) and Bonferroni’s multiple comparison test. The effect of repeated injections of MMP-2/9 inhibitor on allodynia and hyperalgesia was also shown as the area under the curve (AUC, Figure 7). To evaluate the AUC the Trapezoidal and Simpson’s Rules, Pharmacologic Calculation System, version 4.0-03/11/86 was used [86]. Using the GraphPad Prism software, we performed a correlation analysis between the behavioral responses and sympathetic fiber sprouting, as this was the only fiber population which changed following administration of the MMP-2/9 inhibitor. The analysis was done on each behaviorally tested rats from which the skin tissue was collected for the immunohistochemical component.

## Competing interests

The authors declare they have no competing interests.

## Authors’ contributions

MO, GL and SA designed all experimental protocols described in this manuscript. MO performed the behavioral experiments, western blot, zymography as well as the writing of the initial draft of the manuscript. GL performed behavioral experiments and immunohistochemistry. ARdS and ACC provided supervision for data analysis, study direction, image acquisition, manuscript design and revisions. All authors have read and approved the final draft of this manuscript.
