# Correction: Inhibition of endogenous NGF degradation induces mechanical allodynia and thermal hyperalgesia in rats

**DOI:** 10.1186/1744-8069-9-55

**Published:** 2013-11-26

**Authors:** Maria Osikowicz, Geraldine Longo, Simon Allard, A Claudio Cuello, Alfredo Ribeiro-da-Silva

**Affiliations:** 1 Department of Pharmacology and Therapeutics, McGill University, 3655 Prom Sir-William-Osler, Montreal, QC H3G 1Y6, Canada; 2 Department of Anatomy and Cell Biology, McGill University, Montreal, QC H3A 2B2, Canada; 3 Department of Neurology and Neurosurgery, McGill University, Montreal, QC H3A 2B4, Canada

## Correction

It was brought to my attention that the representative bands on Figure 1A, of this work [1] are from a different experiment than the one described and do not represent the quantified data. The quantification and bar graphs are correct, but the representative bands should be replaced by the ones from the corresponding experiment. The error was made during figure preparation. We apologize for the complication and thank you for your help on this issue. The attached file should replace the one that was published. The Figure legend (reproduced below) stays the same.

**Figure 1:**
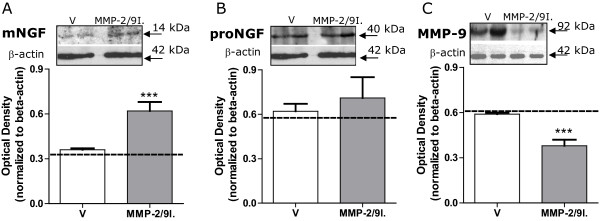
**MMP-2/9 inhibitor administration altered the protein levels of NGF and MMP-9 in the skin of naive rats.** Western blot analysis performed on the glabrous skin samples following 14 days of repeated MMP-2/9 inhibitor (MMP-2/9I.; 20 μg, i.pl.) administration showed a significant increase in the protein levels of mature NGF (mNGF) **(A)**, with no influence on the protein level of the precursor proNGF **(B)**. Repeated administration of MMP-2/9I. (20 μg, i.pl.) significantly reduced the protein levels of MMP-9 **(C)** in the glabrous skin. Examples of representative western blots are presented in the upper panels **A**, **B** and **C**. The densitometry results are presented as the mean ± SEM from all samples. Inter-group differences were analyzed by ANOVA with a Bonferroni’s multiple comparison test. ***p < 0.001 indicates a significant difference as compared to glabrous skin sample of chronic vehicle-treated (V) naive rats. The interrupted line on the graphs indicates protein analyses for mNGF, proNGF or MMP-9 in the contralateral glabrous skin of chronic MMP-2/9I.-treated naïve rats.
